# Predicting out of intensive care unit cardiopulmonary arrest or death using electronic medical record data

**DOI:** 10.1186/1472-6947-13-28

**Published:** 2013-02-27

**Authors:** Carlos A Alvarez, Christopher A Clark, Song Zhang, Ethan A Halm, John J Shannon, Carlos E Girod, Lauren Cooper, Ruben Amarasingham

**Affiliations:** 1 School of Pharmacy – Department of Pharmacy Practice, Texas Tech University Health Sciences Center, 5920 Forest Park Rd, Dallas, TX 75235, USA; 2 Parkland Center for Clinical Innovation, 6300 Harry Hines Blvd, Suite 265, Mailstop 83020, Dallas, TX 75235, USA; 3 Department of Clinical Sciences – Biostatistics Division, University of Texas Southwestern Medical Center, 5323 Harry Hines Blvd, Dallas, TX 75390, USA; 4 Department of Internal Medicine – Division of General Internal Medicine, Department of Clinical Sciences – Division of Outcomes and Health Services Research, University of Texas Southwestern Medical Center, 5323 Harry Hines Blvd, Dallas, TX 75390, USA; 5 Department of Internal Medicine – Division of Respiratory and Critical Care Medicine, Parkland Health and Hospital System – Division of Medical Affairs, University of Texas Southwestern Medical Center, 5201 Harry Hines Blvd, Dallas, TX 75235, USA; 6 Department of Internal Medicine – Division of Respiratory and Critical Care Medicine, University of Texas Southwestern Medical Center, 5323 Harry Hines Blvd, Dallas, TX 75390, USA

**Keywords:** Cardiopulmonary arrest, Forecasting, Medical informatics, Models, Statistical, Medicine, Intensive care units

## Abstract

**Background:**

Accurate, timely and automated identification of patients at high risk for severe clinical deterioration using readily available clinical information in the electronic medical record (EMR) could inform health systems to target scarce resources and save lives.

**Methods:**

We identified 7,466 patients admitted to a large, public, urban academic hospital between May 2009 and March 2010. An automated clinical prediction model for out of intensive care unit (ICU) cardiopulmonary arrest and unexpected death was created in the derivation sample (50% randomly selected from total cohort) using multivariable logistic regression. The automated model was then validated in the remaining 50% from the total cohort (validation sample). The primary outcome was a composite of resuscitation events, and death (RED). RED included cardiopulmonary arrest, acute respiratory compromise and unexpected death. Predictors were measured using data from the previous 24 hours. Candidate variables included vital signs, laboratory data, physician orders, medications, floor assignment, and the Modified Early Warning Score (MEWS), among other treatment variables.

**Results:**

RED rates were 1.2% of patient-days for the total cohort. Fourteen variables were independent predictors of RED and included age, oxygenation, diastolic blood pressure, arterial blood gas and laboratory values, emergent orders, and assignment to a high risk floor. The automated model had excellent discrimination (c-statistic=0.85) and calibration and was more sensitive (51.6% and 42.2%) and specific (94.3% and 91.3%) than the MEWS alone. The automated model predicted RED 15.9 hours before they occurred and earlier than Rapid Response Team (RRT) activation (5.7 hours prior to an event, p=0.003)

**Conclusion:**

An automated model harnessing EMR data offers great potential for identifying RED and was superior to both a prior risk model and the human judgment-driven RRT.

## Background

Out of intensive care unit (ICU) cardiac arrests and unexpected deaths are common despite evidence that patients often show signs of clinical deterioration hours in advance [1-4]. This has prompted national organizations to recommend the implementation of rapid response teams (RRTs) as a strategy to prevent hospital deaths [5]. Such recommendations were made despite conflicting evidence regarding the benefits of RRTs [3,6-10]. Some have speculated that the indeterminate benefit of RRTs is due to insufficiently predictive activation criteria and poor response time by clinical staff [11]. Early warning systems have been developed to identify deteriorating patients using readily available clinical information [12]. However, these early warning systems may not be adequate because they 1) require monitoring and activation by often overburdened clinical staff, 2) fail to systematically monitor all patients, and 3) demonstrate only modest accuracy identifying which patients are at risk of out of ICU cardiopulmonary arrest and death. Early warning systems that are timely, accurate, automated, and comprehensive in their surveillance are needed.

The increasing use of electronic medical records (EMR) in health care makes the use of computerized prediction models possible. These models could represent powerful avenues to identify patients at high risk of adverse events [13,14]. Though a few studies have examined the accuracy of clinical automation to identify patients at risk of clinical deterioration, they retain limited utility since they do not fully harness the EMR, produce no actionable alerts, define primary outcomes differently, and do not allow for monitoring patients in real time [15,16].

This study sought to 1) derive and validate an automated prediction model based on near real-time EMR data to identify patients at high risk of out of ICU resuscitation events and death (RED), 2) compare the test operating characteristics of the new automated model to the previously published Modified Early Warning Score (MEWS) [12] and human judgment-activated institutional RRT, and 3) determine if the automated model detected RED events sooner than the human judgment activated RRT.

## Methods

### Setting and patient population

The automated prediction model was constructed using data from adult patients admitted to Parkland Hospital, a large urban academic hospital in Dallas, TX, between May 18, 2009 and March 31, 2010. Patients were included in the study if they were admitted to the internal medicine ward from either the emergency department (ED) or outpatient clinics. Additionally, patients were included if they were admitted to the ICU from the ED. Patients were excluded if they were directly admitted to the surgical floor or obstetrics or had a do not resuscitate (DNR) order at admission. However, any hospital patient-days prior to a patient consenting to a DNR order were included. To determine if early collection of data was predictive of events, all variables included in the automated model were obtained from the previous calendar day defined as time period between 12:00 AM and 11:59 PM. Therefore, events that occurred on the first day of each hospitalization were excluded. We also excluded any data within one hour of an event to make sure the model did not include factors that were early signs of resuscitation care. Patient-days that occurred after an event were excluded. The research protocol was approved by The University of Texas Southwestern Institutional Review Board (IRB) which concluded that the research presented no more than minimal risk of harm to subjects. Therefore, the IRB waived the need for informed consent.

### Outcome variables

The primary outcome variable was defined as resuscitation events or death (RED). Resuscitation events were defined as out of ICU hospital codes and unplanned transfers to the ICU. Hospital codes included cardiopulmonary arrests (CPA) and acute respiratory compromise (ARC) events, regardless of location, except those that occur in the ICU for ICU length of stays >24 hours. CPA was defined as an event in which chest compressions and/or defibrillation are delivered, and an ARC event was defined as an event requiring emergency assisted ventilation [17]. These events were identified electronically through the hospital’s internal registry which is structured on the American Heart Association’s Get With The Guidelines – Resuscitation national registry, formally known as the National Registry of Cardiopulmonary Resuscitation [17]. This registry collects data on in-hospital resuscitation events from hospitals across the United States to provide feedback on an institution’s resuscitation practices and patient outcomes. Unplanned ICU transfers included any transfers from the internal medicine ward or ED to a medical or cardiac ICU requiring an ICU length of stay >24 hours. We used unplanned ICU transfer in the definition of a RED event because these patients were in critical condition and would have a high likelihood of CPA or death had the transfer not occurred. There are no elective admissions to the ICU at this institution. Unexpected death was defined as: 1) an in-hospital death that occurred on the medical ward; or 2) death that occurred in patients transferred to a medical or cardiac ICU team with an ICU length of stay <24 hours. Patient death and transfers to the medical or cardiac ICU were identified electronically in the hospital’s EMR. The date and time of bedside RRT activation was extracted from the hospital’s systematic log of all RRT calls. Data used to predict the primary outcome were extracted from the previous calendar day.

### Predictor variables

We developed a conceptual model of RED events based on a comprehensive review of the literature and expert clinical opinion. Candidate predictor variables for the automated model were those extractable from the hospital EMR (EPIC Systems Corporation, Verona, WI). Data from the previous 24 hours calendar day were used to determine the daily risk. Potential predictor variables included the most abnormal laboratory value or vital sign in the 24 hours period between 12:00 AM to 11:59 PM on each hospital day. We also examined other possible indicators of impending RED events such as STAT physician orders and medications. Medications of interest were those thought to increase risk of serious adverse events according to the Institute for Safe Medication Practices (ISMP). The MEWS is a previously published risk score based on the number and degree of vital sign and level of consciousness (LOC) abnormalities (Additional file 1: Appendix A) [12]. We determined LOC using a text-processing algorithm to read the free text in nursing notes. Finally, we postulated that patients who were more ill or unstable in subtle, hard-to-measure ways could be preferentially admitted to certain non-ICU medical floors, so we classified medicine wards accounting for the top 15% of RED as “high risk floors.”

### Derivation and validation of the automated prediction model

The automated model was constructed in stages. First, the total cohort was randomly split into derivation (50%) and validation (50%) subsamples. We constructed the final model using the derivation cohort. Second, recursive partitioning was used to identify significant cut-points in continuous candidate variables that were associated with an increased rate of RED events. Third, candidate predictors of RED events were identified using univariate logistic regression. Continuous variables were examined for nonlinear effects by testing the contributions of spline functions and variable transformations. Fourth, candidate variables significant at p ≤ 0.20 were entered into a multivariate logistic regression model. Final model variables were selected on the basis of conceptual and statistical significance (p ≤0.05). The unit of analysis was in patient-days.

The model based on the derivation dataset was validated by comparing its performance in the validation sample. Model discrimination was assessed with the c-statistic and calibration with the Hosmer-Lemeshow goodness-of-fit test [18]. Using cut-points determined by the derivation subsample, five risk categories were created on quintiles of predicted risk and graphically assessed in the validation sample. To account for within patient correlation, we used robust variance–covariance matrix estimators for computing standard errors for model coefficients.

Prior to model development, we assessed all variables for missing values. For categorical and continuous variables with less than 2% missing data, a missing category was created, and the event rate was compared with and pooled into the most appropriate reference group. For categorical and continuous variables that had greater than 2% missing data and were not measured from one day to the next, a “never measured” category was created and risk was compared to the other categories or cut-points and pooled into the appropriate reference group. Documentation by exception is a common approach in the predictive model literature [13,14,19,20].

We determined relative contribution of each predictor to RED events by examining the marginal increase in the model chi-square accounted for by each predictor as it was added and removed from the final automated model [21,22].

### Comparing performance of the automated model to the MEWS

Patients were classified to be at risk of RED events at a probability threshold of 4% as determined by the automated model. Since the baseline risk for RED events was assumed to be 1%, we considered a four times greater than average risk an important threshold for concern. Variables used to calculate the MEWS were obtained in the previous calendar day between 12:00 AM to 11:59 PM. If a patient experienced a RED event, data from the previous calendar day and those up to one hour prior to the event were used to calculate the MEWS. A MEWS of ≥5 was considered the critical threshold based on the literature [12]. Sensitivity, specificity, positive predictive value, and negative predictive value were determined for both the automated model and the MEWS. The test operating characteristics of the automated model and the MEWS were compared using the c-statistic. Confidence intervals were constructed for the c-statistics at the 95% level [18].

### Comparing performance of the automated model to the institutional RRT

The institutional RRT is deployed when one or more of the following is present in a patient: 1) heart rate <40 or >130 beats/min, 2) systolic blood pressure <90 mmHg, 3) respiratory rate <8 or >30 breaths/min, 4) partial pressure of oxygen <88% on room air, 5) oxygen requirement >50%, and 6) acute change in mental status. We calculated the sensitivity, specificity, positive predictive value and negative predictive value, along with 95% confidence intervals, for both the automated model and the institutional RRT. Moreover, we evaluated a subgroup of patients that experienced an event who activated the institutional RRT and had a predicted probability of a RED event of 4% by the automated model (model activation). In this subgroup, we aimed to determine the difference in time between model activation and RRT deployment. We also evaluated the time difference between the automated trigger of a RED event (patient’s predicted probability of a RED event exceeds 4%) and RRT deployment, regardless of an event. Our hypothesis was that the automated model would detect a patient who had a RED event well in advance of the institutional RRT. We compared this time difference using a paired Student’s *t*-test. Analyses were conducted using STATA statistical software (version 10.0; STATA Corp, College Station, TX) and RTREE [23].

## Results

### Patient characteristics

A total of 7,466 hospitalized patients accounted for 46,974 patient-days. The derivation and validation cohorts were evenly matched across demographic, clinical, provider orders, administered medications and summary variables (Table 1). Mean age was 51.2 in the derivation cohort and 51.4 in the validation cohort, and 56.1% and 54% were male, respectively.

**Table 1:** Cohort characteristics (N=46,974 patient-days)

	**Derivation (n=23,127)**	**Validation (n=23,847)**
Number of Patients:	3624	3792
RED events ^*a*^, n(%)	298 (1.3)	287 (1.2)
***Demographics***		
Age, mean (SD)	50.5 (14.6)	51 (14.8)
Male, n(%)	2,062 (56.1)	2,049 (54)
***Vital Signs, mean (SD)***		
Temperature (°F)	98.1 (1.6)	98.3 (1.8)
Systolic Blood Pressure (mm Hg)	139.8 (25.0)	139.6 (24.6)
Respirations per minute	21 (7.3)	20.7 (6.5)
Pulse per minute	91.3 (19.0)	92.1 (18.9)
Diastolic Blood Pressure (mm Hg)	83.8 (15.1)	83.9 (15.4)
SpO2 (%)	95.6 (5.1)	95.6 (5.0)
***Laboratory Findings, mean (SD)***		
Platelets (10^3^ cells/mm^3^)	242.3 (125.6)	237.8 (122.6)
Potassium (mEq/L)	4.0 (0.6)	4.0 (0.6)
Glucose (mg/dL)	128.6 (76.5)	127.8 (73.9)
Hematocrit (g/dL)	32.4 (6.8)	32.4 (6.7)
Creatinine (mg/dL)	2 (2.9)	1.8 (2.7)
White Blood Cell Count (10^3^ cells/mm^3^)	8.6 (9.5)	8.6 (7.3)
Total Bilirubin (mg/dL)	1.5 (4.0)	1.5 (3.6)
Sodium (mEq/L)	135.8 (4.2)	135.8 (4.4)
Arterial pH	7.4 (0.1)	7.4 (0.1)
Arterial pCO2 (mm Hg)	37.5 (13.1)	38.9 (13.5)
AST (U/L)	95.6 (417.2)	74.5 (211.0)
Anion Gap	10.6 (3.9)	10.5 (4.0)
Albumin (g/dL)	3.2 (0.7)	3.2 (0.7)
B-type Natriuretic Peptide (pg/mL)	7,291.6 (14455.7)	6,357.2 (13019.8)
Thyroid Stimulating Hormone (μIU/mL)	5.4 (21.6)	4.4 (27.0)
Estimated Glomerular Filtration Rate (mL/min/1.73 m^2^)	50.5 (17.7)	50.7 (17.3)
Level of Consciousness	0.1 (0.4)	0.1 (0.3)
***Provider Orders, n(%)***		
Bilevel positive airway pressure	122 (0.5)	178 (0.7)
Arterial Blood Gas	1,436 (6.2)	1,509 (6.3)
Troponin I	3,725 (16.1)	3,804 (16.0)
Electrocardiogram	4,996 (21.6)	5,143 (21.6)
Electroencephalogram	87 (0.4)	115 (0.5)
Telemetry	1,765 (7.6)	1,787 (7.5)
Stat order # 1 ^*b*^	1,704 (7.4)	1,902 (8.0)
Stat order # 2 ^*c*^	2,476 (10.7)	2,614 (11.0)
***Administered Medications, n(%)***		
Institute of Safe Medication Practice High Alert Medication ^*d*^	9,257 (40.0)	9,554 (40.1)
Systemic Steroids ^*e*^	758 (3.3)	808 (3.4)
Sodium Bicarbonate	423 (1.8)	427 (1.8)
Lactulose or Rifaxamin	535 (2.3)	634 (2.7)
Antidote medication ^*f*^	308 (1.3)	341 (1.4)
More than one nephrotoxic agent taken concurrently ^*g*^	3,580 (15.5)	3,502 (14.7)
More than one antibiotic agent taken concurrently ^*h*^	2,549 (11.0)	2,611 (11.0)
Intravenous fluid bolus	1,270 (5.5)	1,329 (5.6)
Stat acute coronary syndrome medications ^*i*^	2,436 (10.5)	2,276 (9.5)
Stat seizure abatement medications ^*j*^	1,069 (4.6)	1,234 (5.2)
***Summary Variables***		
MEWS, mean (SD) ^*k*^	2.2 (1.5)	2.2 (1.5)
High Risk Floor Assignment, n(%) ^*l*^	2,369 (10.2)	2,348 (9.9)

*a* Resuscitation events and death; *b* Stat orders for head magnetic resonance imaging or computed tomography scan, chest plain film x-rays or computed tomography scan, and abdominal ultrasonography; *c* Stat orders for complete blood count, bilevel positive airway pressure, arterial blood gas, troponin, electroencephalogram and electrocardiogram; *d* antithrombotic agents, chemotherapeutic agents, epidural or intrathecal route of administration, hypoglycemic, magnesium sulfate injection, hypertonic sodium chloride, colchicine injection, and methotrexate oral; *e* IV or oral prednisone, methylprednisolone, prednisolone, fludricortisone and hydrocortisone; *f* Protamine, naloxone, phytonadione, flumazenil; *g* Systemic amphotericin B (lipid and conventional), systemic aminoglycosides, scheduled daily non-steroidal anti-inflammatories, IV vancomycin, angiotensin converting enzyme inhibitors, angiotensin II receptor blockers, cyclosporine, tacrolimus, loop diuretics, penicillin antibiotics, acyclovir; *h* Any aminoglycoside, penicillin, cephalosporin, vancomycin, sulfa antibiotic, linezolid, doxycycline, tigecycline, fluoroquinolone, aztreonam, carbapenem, daptomycin, and nitrofurantoin; *i* IV metoprolol, sublingual or IV nitroglycerin and aspirin given concurrently; *j* intramuscular lorazepam or diazepam, phenobarbital, fosphenytoin, and phenytoin; *k* elements consists of systolic blood pressure, heart rate, respiratory rate, temperature, and level of consciousness; *l* includes floors that comprise the top 15% of events.

### Primary outcomes and predictors of RED events

Major clinical deterioration occurred in 1 in 100 admissions (1.3% and 1.2% of hospitalizations in the derivation and validation cohorts). The univariate predictors of RED events are shown in Table 2 and included: older age (>54 years), abnormal vital signs (temperature >99.5, respiratory rate >24 bpm, DBP >125 mm/Hg), abnormal laboratory values (e.g., potassium >5.1 mEq/L, glucose >600 mg/dL, sodium <128 mEq/L), abnormal arterial blood gas (ABG) results (pCO2 ≤22 mmHg or pCO2 >70 mmHg), STAT physician orders (CBC order, electrocardiogram order, ABG order), high risk floor assignment, high alert medication orders (ISMP high alert medications, antidote medications, IV fluid bolus), level of consciousness, and the MEWS score.

**Table 2:** Univariate predictors of RED events (N= 23,127 patient days)

**Variable**	**Derivation**	**Events # (%)**	**Odds Ratio (95% CI)**	**p**
RED events ^*a*^	298 (1.3)			
age > 54	9,217 (39.9)	145 (1.6)	1.4 (1.14 - 1.81)	0.002
**Vitals, N(%)**				
Temperature (°F)				
≤99.5 (ref.)	20,157 (87.2)	229 (1.1)	--	--
>99.5	2,970 (12.8)	69 (2.3)	2.1 (1.58 - 2.72)	<0.001
Systolic Blood Pressure (mm Hg)				
≤125 (ref.)	7,122 (30.8)	77 (1.1)	--	--
>125	16,005 (69.2)	221 (1.4)	1.3 (0.99 - 1.66)	0.063
Respirations per minute				
≤24 (ref.)	21,713 (93.9)	192 (0.9)	--	--
>24	1,414 (6.1)	106 (7.5)	9.1 (7.12 - 11.59)	<0.001
Pulse per minute				
≤134 (ref.)	22,464 (97.1)	240 (1.1)	--	--
>134	663 (2.9)	58 (8.8)	8.9 (6.59 - 11.96)	<0.001
Diastolic Blood Pressure (mm Hg)				
≤120 (ref.)	22,507 (97.3)	265 (1.2)	--	--
>120	620 (2.7)	33 (5.3)	4.7 (3.26 - 6.84)	<0.001
spO2 (%)				
≤86	319 (1.4)	25 (7.8)	7.0 (4.59 - 10.74)	<0.001
>86 (ref.)	22,808 (98.6)	273 (1.2)	--	--
**Laboratory, N(%)**				
Platelets (10^3^ cells/mm^3^)				
<100	1959 (8.5)	32 (1.6)	1.3 (0.90 - 1.89)	0.158
≥100 (ref.)	21,168 (91.5)	266 (1.3)	--	--
Potassium (mEq/L)				
≤2.9	155 (0.7)	9 (5.8)	5.4 (2.74 - 10.76)	<0.001
2.9 - 5.1 (ref.)	21,811 (94.3)	245 (1.1)	--	--
>5.1	1,161 (5.0)	44 (3.8)	3.5 (2.50 - 4.81)	<0.001
Glucose (mg/dL)				
≤250 (ref.)	22,219 (96.0)	276 (1.2)	--	--
250 – 600	825 (3.6)	17 (2.1)	1.7 (1.02 - 2.74)	0.042
>600	83 (0.4)	5 (6.0)	5.1 (2.05 - 12.69)	<0.001
Hematocrit (g/dL)				
≤48 (ref.)	22,925 (99.1)	288 (1.3)	--	--
> 48	202 (0.9)	10 (5.0)	4.1 (2.15 - 7.81)	<0.001
Creatinine				
≤1.5 (ref.)	17,937 (77.6)	201 (1.1)	--	--
>1.5	5,190 (22.4)	97 (1.9)	1.7 (1.32 - 2.15)	<0.001
White Blood Cell Count (10^3^ cells/mm^3^)				
≤11 (ref.)	19,225 (83.1)	204 (1.1)	--	--
>11	3,902 (16.9)	94 (2.4)	2.3 (1.80 - 2.95)	<0.001
Total Bilirubin (mg/dL)				
≤1.7 (ref.)	22,172 (95.9)	271 (1.2)	--	--
>1.7	955 (4.1)	27 (2.8)	2.4 (1.57 - 3.51)	<0.001
Sodium (mEq/L)				
≤128	983 (4.2)	29 (3.0)	2.5 (1.69 - 3.69)	<0.001
128 - 143 (ref.)	21,808 (94.3)	262 (1.2)	--	--
>143	336 (1.5)	7 (2.1)	1.7 (0.82 - 3.74)	0.148
pH				
≤7.22	53 (0.2)	11 (20.8)	20.8 (10.60 - 40.80)	<0.001
>7.22 (ref.)	23,074 (99.8)	287 (1.2)	--	--
pCO2 (mm Hg)				
≤22	78 (0.3)	12 (15.4)	14.8 (7.89 - 27.62)	<0.001
22 - 70 (ref.)	23,020 (99.5)	280 (1.2)	--	--
>70	29 (0.2)	6 (20.7)	21.2 (8.56 - 52.43)	<0.001
AST (U/L)				
≤250 (ref.)	22,826 (98.7)	279 (1.2)	--	--
>250	301 (1.3)	19 (6.3)	5.4 (3.37 - 8.79)	<0.001
Anion Gap				
≤16 (ref.)	21,791 (94.2)	236 (1.1)	--	--
>16	1,336 (5.8)	62 (4.6)	4.4 (3.34 - 5.91)	<0.001
Albumin (g/dL)				
< 3.5	2,779 (12.0)	54 (1.9)	1.6 (1.21 - 2.20)	0.001
≥3.5 (ref.)	20,348 (88.0)	244 (1.2)	--	--
B-type natriuretic peptide (pg/mL)				
≤100 (ref.)	21,803 (94.3)	257 (1.2)	--	--
>100	1,324 (5.7)	41 (3.1)	2.7 (1.92 - 3.74)	<0.001
Thyroid Stimulating Hormone (μIU/mL)				
≤0.4	60 (0.3)	3 (5.0)	4.1 (1.27 - 13.10)	0.018
0.4 - 5.4	610 (2.6)	7 (1.2)	0.9 (0.42 - 1.91)	0.784
>5.4	83 (0.4)	3 (3.6)	2.9 (0.91 - 9.26)	0.071
Not measured (ref.)	22,374 (96.7)	285 (1.3)	--	--
Estimated glomerular filtration rate (mL/min/1.73 m^2^)				
≤30	3,009 (13.0)	57 (1.9)	1.6 (1.19 - 2.13)	0.002
>30 (ref.)	20,118 (87.0)	241 (1.2)	--	--
**Physician Orders, N(%)**				
Bilevel positive airway pressure	122 (0.5)	11 (9.0)	7.8 (4.18 - 14.73)	<0.001
Arterial Blood Gas	1,436 (6.2)	104 (7.2)	8.7 (6.77 - 11.05)	<0.001
Troponin I	3,725 (16.1)	130 (3.5)	4.1 (3.28 - 5.22)	<0.001
Electrocardiogram	4,996 (21.6)	183 (3.7)	6.0 (4.71 - 7.54)	<0.001
Electroencephalogram	87 (0.4)	1 (1.2)	0.9 (0.12 - 6.41)	0.908
Telemetry	1,765 (7.6)	36 (2.0)	1.7 (1.18 - 2.38)	0.004
Stat order # 1 ^*b*^	1,704 (7.4)	45 (2.6)	2.3 (1.65 - 3.13)	<0.001
Stat order # 2 ^*c*^	2,476 (10.7)	70 (2.8)	2.6 (1.99 - 3.42)	<0.001
**Administered Medications, N(%)**				
Institute of Safe Medication Practice High Alert Medication ^*d*^	9,257 (40.0)	162 (1.8)	1.8 (1.43 - 2.26)	<0.001
Systemic Steroids ^*e*^	758 (3.3)	13 (1.7)	1.4 (0.77 - 2.37)	0.292
Sodium Bicarbonate	423 (1.8)	16 (3.8)	3.1 (1.87 - 5.22)	<0.001
Lactulose or Rifaxamin	535 (2.3)	12 (2.2)	1.8 (1.00 - 3.21)	0.051
Antidote medication ^*f*^	308 (1.3)	14 (4.6)	3.8 (2.18 - 6.54)	<0.001
More than one nephrotoxic agent taken concurrently ^*g*^	3,580 (15.5)	60 (1.7)	1.4 (1.04 - 1.84)	0.026
More than one antibiotic agent taken concurrently ^*h*^	2,549 (45.3)	46 (1.8)	1.5 (1.08 - 2.04)	0.015
Intravenous fluid bolus	1,270 (5.5)	55 (4.3)	4.0 (2.99 - 5.43)	<0.001
Stat acute coronary syndrome medications ^*i*^	2,436 (10.5)	43 (1.8)	1.4 (1.04 - 1.99)	0.028
Stat seizure abatement medications ^*j*^	1,069 (4.6)	31 (2.9)	2.4 (1.67 - 3.55)	<0.001
**Summary Variables**				
MEWS, Mean (SD) ^*k*^	2.28 (1.5)		1.6 (1.54 - 1.70)	<0.001
Level of Consciousness				
0 – Alert	7,580 (32.8)	156 (2.1)	3.1 (2.41 - 4.00)	<0.001
1 – Responds to voice/new confusion/restlessness	543 (2.3)	23 (4.2)	6.5 (4.12 - 10.37)	<0.001
2 – Responds to pain	102 (0.4)	6 (5.9)	9.2 (3.95 - 21.55)	<0.001
3 – Unresponsive	32 (0.1)	13 (40.6)	101.1 (48.59 - 210.20)	<0.001
Not measured (ref.)	14,870 (64.3)	100 (0.7)	--	--
High Risk Floor Assignment, N(%) ^*l*^	2,369 (10.2)	158 (6.7)	10.5 (8.35 - 13.27)	<0.001

a Resuscitation events and death; b Stat orders for head magnetic resonance imaging or computed tomography scan, chest plain film x-rays or computed tomography scan, and abdominal ultrasonography; c Stat orders for complete blood count, bilevel positive airway pressure, arterial blood gas, troponin, electroencephalogram and electrocardiogram; d antithrombotic agents, chemotherapeutic agents, epidural or intrathecal route of administration, hypoglycemic, magnesium sulfate injection, hypertonic sodium chloride, colchicine injection, and methotrexate oral; e IV or oral prednisone, methylprednisolone, prednisolone, fludricortisone and hydrocortisone; f Protamine, naloxone, phytonadione, flumazenil; g Systemic amphotericin B (lipid and conventional), systemic aminoglycosides, scheduled daily non-steroidal anti-inflammatories, IV vancomycin, angiotensin converting enzyme inhibitors, angiotensin II receptor blockers, cyclosporine, tacrolimus, loop diuretics, penicillin antibiotics, acyclovir; h Any aminoglycoside, penicillin, cephalosporin, vancomycin, sulfa antibiotic, linezolid, doxycycline, tigecycline, fluoroquinolone, aztreonam, carbapenem, daptomycin, and nitrofurantoin; i IV metoprolol, sublingual or IV nitroglycerin and aspirin given concurrently; j intramuscular lorazepam or diazepam, phenobarbital, fosphenytoin, and phenytoin; k elements consists of systolic blood pressure, heart rate, respiratory rate, temperature, and level of consciousness; l includes floors that comprise the top 15% of events.

In the multivariable analysis, 14 variables were independent predictors of RED events including: age >54 years, abnormal vital signs (DBP >120 mmHg, SpO_2_ ≤86%) abnormal laboratory values (AST >250 U/L, white blood cell count >11 × 10^3^ cells/mm^3^, platelets <100 × 10^3^ cells/mm^3^, potassium >5.1 mEq/L), abnormal ABG results (pCO2 ≤22 mmHg or pCO2 >70 mmHg), physician orders for an ABG, electrocardiogram, STAT orders for head computed tomography (CT) or magnetic resonance imaging, chest CT, abdominal ultrasound, and chest x-rays, high risk floor assignment and summary MEWS score (Table 3). The strongest individual indicators of RED events were: abnormal ABG results and high risk floor assignment.

**Table 3:** Multivariate predictors of RED events (derivation cohort, N= 23,127)

**Variables**	**Odds Ratio (95% CI)**	**P Value**
***Demographics/Laboratory/Vital Signs***		
Age >54 years	1.59 (1.24 - 2.04)	<0.001
SpO2 (%) (min) ≤86	1.73 (1.04 - 2.88)	0.03
Diastolic BP (mm Hg) (max) >120	3.17 (1.16 - 8.66)	0.03
AST (U/L) (min) >250	2.89 (1.62 - 5.17)	<0.001
pCO2 (mm Hg) (max) ≤22	3.72 (1.64 - 8.40)	0.002
pCO2 (mm Hg) (max) >70	4.61 (1.84 - 11.56)	0.001
WBC (10^3^ cells/mm^3^) (min) >11	1.36 (1.03 - 1.79)	0.03
Platelets (10^3^ cells/mm^3^) (min) <100	1.76 (1.18 - 2.64)	0.01
Potassium (mEq/dL) (max) >5.1	1.77 (1.22 - 2.56)	0.003
***Orders***		
Arterial Blood Gas	2.08 (1.52 - 2.85)	<0.001
Electrocardiogram	2.05 (1.54 - 2.73)	<0.001
Stat Physician Order ^*a*^	2.20 (1.53 - 3.16)	<0.001
***Summary Variable***		
High Risk Floor Assignment ^*b*^	5.71 (4.34 - 7.51)	<0.001
MEWS ^*c*^	1.36 (1.28 - 1.44)	<0.001
**Derivation: 0.87 (0.85 - 0.89) - 298 events**		
**Validation: 0.85 (0.82 - 0.87) - 287 events**		

*a* Stat orders for head magnetic resonance imaging or computed tomography scan, chest plain film x-rays or computed tomography scan, and abdominal ultrasonography; *b* includes floors that comprise the top 15% of events; *c* elements consists of systolic blood pressure, heart rate, respiratory rate, temperature, and level of consciousness.

### Performance of the automated model

**Figure 1:**
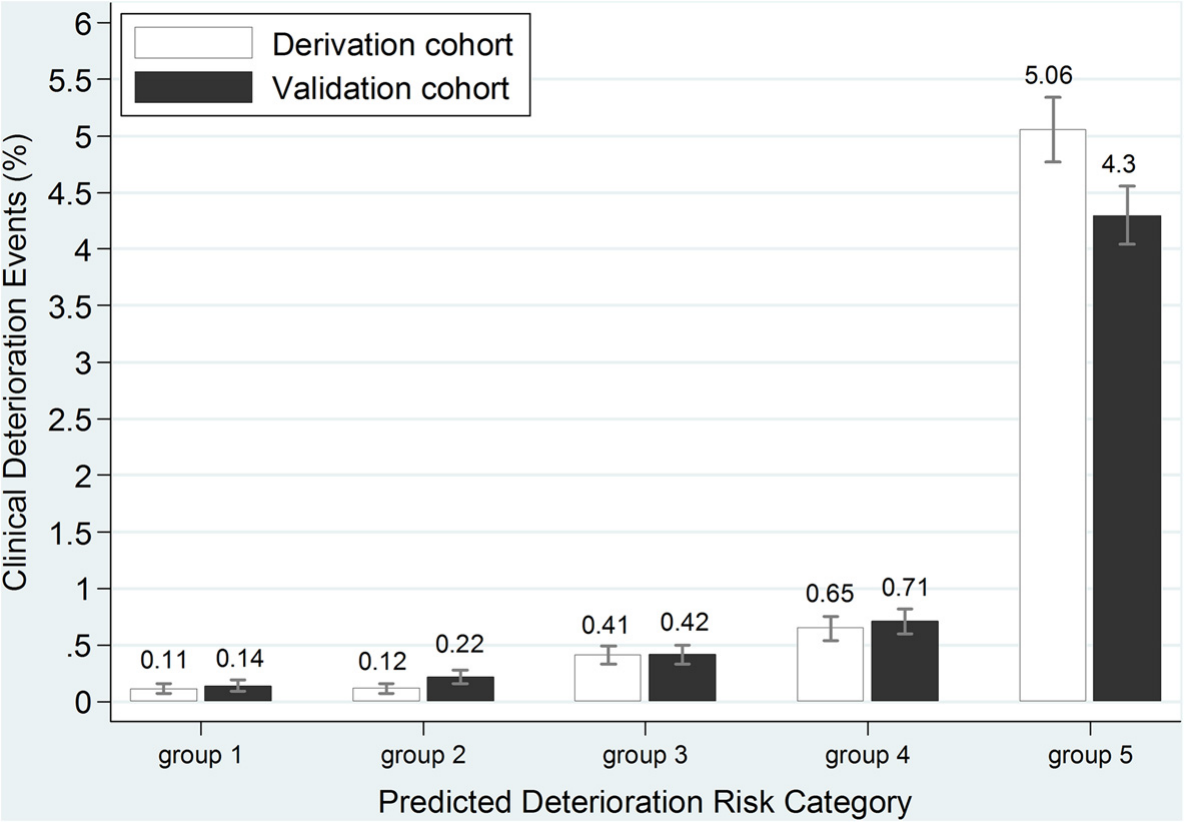
**Observed rates of RED events stratified by quintiles of risk in the automated model.** Legend: Group 1 is the lowest quintile of risk and group 5 is the highest quintile of risk. The Figure shows comparable performance in the derivation (white bars) and validation (black bars) samples.

The final automated model had good discrimination in both the derivation and validation dataset with a c-statistic of 0.87 (95% CI 0.85-0.89) and 0.85 (95% CI 0.82 - 0.87), and was well-calibrated (Hosmer Lemeshow test p=0.12). It also stratified patients across a wide spectrum of risk from 0.14% in the lowest quintile to 4.3% in the highest one (Figure 1). The principal influencing variables in the automated model as assessed by the uniquely attributable chi-square were high risk floor assignment (37.9%) followed by the MEWS (25.5%), demographics, laboratory and vital signs (18.2%), and physician orders (18.4%).

### Comparing the performance of the automated model performance and the MEWS

**Figure 2:**
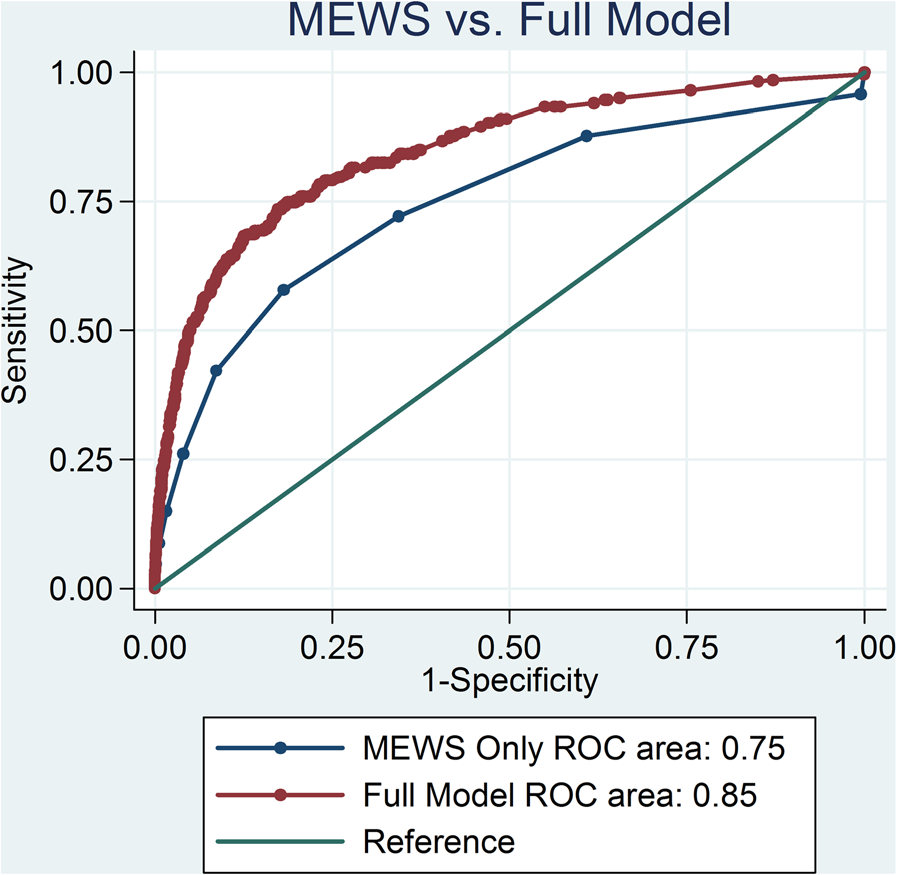
Comparing Receiver Operator Characteristic curve performance for final automated model versus the MEWS.

The automated model was both more sensitive (51.6% and 42.2%) and specific (94.3% and 91.3%) than the MEWS. The positive predictive value (PPV) of the automated model was superior to the MEWS (10% and 5.6%). The negative predictive values (NPV) were similar (99.4% and 99.2%). The automated model performed significantly better than the MEWS with a c-statistic of 0.85 (95% CI 0.82 - 0.87) compared to a c-statistic of 0.75 (95% CI 0.71 - 0.78) (Figure 2).

### Comparing the performance of the automated model to the RRT calls

The RRT was activated for 357 of eligible study patients as part of usual care during the study period. The automated model was more sensitive than the RRT (51.6% vs. 25.8%). However, it was slightly less specific than the RRT (98.8% vs. 94.3%). The RRT had a better PPV than the automated model (21% and 10%) and similar NPV (99.1% and 99.4%). The median number of times the automated model flagged patients at risk per day during the study period was 9 and the median number of RRT calls per day was 2.

There were a total of 17 patients who were at risk of RED events by the automated model, where the institutional RRT was deployed and experienced a RED event. The automated model predicted an event 15.9 (±7.7) hours before the actual event occurred compared to the RRT which was called a mean of 8.4 (±8.5) hours prior the actual event (p=0.003). Overall, the automated model also determined a patient to be at risk 5.7 hours (95% CI 3.1-8.3) earlier than the RRT was called for all types of RED events.

## Discussion

We developed and validated a novel, automated model using the EMR for predicting RED events in patients admitted to the hospital. From a statistical perspective, the automated model had excellent discrimination, was well-calibrated, and had outstanding specificity (94.3%) and good sensitivity (51.6%). The automated model also had better discrimination, sensitivity and specificity than the previously published MEWS. From a practical standpoint, the model identified patients destined to have RED event on average 16 hours (or more than one nursing shift) before they actually experienced a major clinical event. Further, the automated model was able to accurately predict RED events using information obtained from the previous 24 hours. Together with its ability to screen all patients systematically and automatically, low false positive rate, and advance notice, the automated model appears to provide both accurate and actionable intelligence.

Since the growing standard of care is to use the RRTs to meet this goal, we were particularly interested in the more practical comparison of the new model to the human or manually activated RRT approach used in our hospital. Overall, the automated model had twice the sensitivity of the RRT (51.6% v. 25.8%), demonstrating that computerized surveillance is likely to identify more patients at risk for major adverse events compared to providers’ clinical judgment. The automated model achieved this much higher sensitivity with only a small trade-off in specificity (94.3% v. 98.8%). Perhaps of greatest importance from a patient safety viewpoint, the automated model flagged patients 5.7 hours sooner than the RRT. Accurately identifying patients earlier in of the course of physiological deterioration should be expected to yield greater opportunity for rescue.

The superior performance of the new model likely came from the richer source of information available in the EMR which is unavailable to simpler vital sign based models. In addition, monitoring physician orders for ECG, ABG or other STAT orders appears to be an important predictive measure, perhaps reflecting a physician’s escalating concern about a patient’s stability. Novel variables, such as high risk floor assignment, may be a proxy for nurse staffing ratios, physician team composition, or other unknown system or process-related factors that are associated with increased acuity or risk.

We were somewhat surprised that none of the medication variables were included in the final model, despite looking at many candidate predictors. This result may be due to the administration of antidote medicines that occur late in the process of clinical deterioration. The risk of causing RED events due to use of high risk medicines may be mediated through their effect on vital sign and laboratory abnormalities and partly depend on a patient’s underlying hepatic and renal physiological reserve. There is a need to explore more complex drug interactions and their association with adverse events.

The 1.3% prevalence in this study is similar to that seen in other studies [3,6]. The performance of the MEWS in this study was also consistent with prior reports (c-statistic=0.75), confirming its moderate predictive capabilities [12,15]. Our institution had an RRT call rate similar to those observed elsewhere [24].

Several limitations are worth noting. First, we used retrospective data from a single urban health system to derive and validate our model. While the rate of RED events and RRT calls in this sample is similar to other studies, the generalizability of this model to other patient populations and health systems is unknown and merits further investigation [3]. Second, the derivation and validation of the novel model was done retrospectively, so the next step would be prospective validation ideally in more than one setting. Third, and even more importantly, the ultimate value of the automated model will depend on whether it can realistically be used in real-time and if flagging patients at high risk will change clinical management, improves patient outcomes and/or reduces human surveillance burden. While we hypothesize that earlier warning and proper identification of patients at risk will decrease RED events, this has yet to be shown. Fourth, although the automated model achieves a c-statistic of 0.85, there is a moderate false positive rate. However, given the severity of RED events, we accept the false positive rate in exchange for greater model sensitivity. More work is necessary to prevent the activation of overburdened clinical staff to false alerts. Fifth, there may be some difficulty generalizing “high risk floors”, although, institutions can determine the rate of RED for each floor and establish which areas comprise the top 15% of events. Finally, our model uses data derived from a comprehensive EMR, so it may only be useful in such settings. However, the deployment of integrated EMRs in hospitals has been accelerating greatly due to recent federal investments in health information technology and is expected to continue over the next 5 to 10 years [25-27]. While our model has robust predictive capabilities, we believe employing additional technologies such as natural language processing may further improve prediction. Another area of promise involves more sophisticated adverse drug event detection software to further classify risk and improve prediction of poor hospital outcomes.

## Conclusion

One in 100 hospitalized medical patients experienced RED events, among the most serious of all adverse patient safety outcomes. The novel, EMR-based model we developed was better at predicting these serious adverse events compared to prior risk models and the human judgment based RRT approach. While formal prospective implementation and evaluation of such a computerized RED event risk detection strategy is needed in the form of a controlled trial, this automated prediction model could be a powerful tool in the effort to reduce out of ICU CPA, unplanned transfers to the ICU, and death. Models such as ours may foreshadow higher level “meaningful use” of EMRs to improve inpatient outcomes.

## Abbreviations

ABG: Arterial blood gas;ARC: Acute respiratory compromise;CBC: Complete blood count;CPA: Cardiopulmonary resuscitation;CT: Computed tomography;DNR: Do not resuscitate;ECG: Electrocardiogram;ED: Emergency department;EMR: Electronic medical record;ICU: Intensive care unit;ISMP: Institute for safe medication practices;IV: Intravenous;LOC: Level of consciousness;MEWS: Modified early warning score;NPV: Negative predicted value;PPV: Positive predicted value;RED: Resuscitation events and death;RRT: Rapid response team

## Competing interest

The authors of this study declare no competing interest with regards to this publication.

## Authors’ contribution

CAC and RA participated in the study concept and design, and acquisition of data. CAA, CAC, EAH, JJS, CEG, SZ and RA participated in the analysis and interpretation of the data. CAA, CAC, LC and RA participated in drafting the manuscript. CAA, CAC, SZ, EAH, JJS, CEG, LC and RA provided critical revisions of the manuscript. CAA, CAC, SZ and RA participated in the statistical analysis of the data. JJS and RA provided administrative, technical and material support for the research. RA provided supervision. All authors read and approved the final manuscript.

## Pre-publication history

The pre-publication history for this paper can be accessed here:

http://www.biomedcentral.com/1472-6947/13/28/prepub

## Supplementary Material

Additional file 1: Appendix A Modified Early Warning Score ^*a*^. Click here for file
