# Supplementary material for: Predicting out of intensive care unit cardiopulmonary arrest or death using electronic medical record data
Source: BMC Med Inform Decis Mak. 2013 Feb 27;13:28. doi: 10.1186/1472-6947-13-28 (PMC3599266; doi:10.1186/1472-6947-13-28)
Supplement: Additional file 1: Appendix A — Modified Early Warning Score a. [file 1472-6947-13-28-S1.docx]

| **Appendix A. Modified Early Warning Score *^a^*** | | | | | | | |
| --- | --- | --- | --- | --- | --- | --- | --- |
| Score | 3 | 2 | 1 | 0 | 1 | 2 | 3 |
| Heart Rate  (beats per minute) |  | ≤40 | 41-50 | 51-100 | 101-110 | 111-129 | ≥130 |
| Systolic Blood Pressure  (mm Hg) | ≤70 | 71-80 | 81-100 | 101-199 |  | ≥200 |  |
| Respiratory Rate  (breaths per minute) |  | ≤8 |  | 9-14 | 15-20 | 21-29 | ≥30 |
| Temperature (°F) |  | <95 |  | 95-101.2 |  | ≥101.3 |  |
| Level of Consciousness |  |  |  | Alert | Responds to voice/new confusion/restlessness | Responds to pain | Unresponsive |
| *^a^*Adapted from Subbe CP, Kruger M, Rutherford P, Gemmel L. Validation of a modified Early Warning Score in medical admissions. QJM. 2001;94(10):521-6. | | | | | | | |
